# Increased Microparticle Production and Impaired Microvascular Endothelial Function in Aldosterone-Salt-Treated Rats: Protective Effects of Polyphenols

**DOI:** 10.1371/journal.pone.0039235

**Published:** 2012-07-10

**Authors:** Natalia López Andrés, Angela Tesse, Véronique Regnault, Huguette Louis, Valérie Cattan, Simon N. Thornton, Carlos Labat, Agustine Kakou, Simon Tual-Chalot, Sébastien Faure, Pascale Challande, Mary Osborne-Pellegrin, M. Carmen Martínez, Patrick Lacolley, Ramaroson Andriantsitohaina

**Affiliations:** 1 Institut National de la Santé et de la Recherche Médicale, U961, Nancy Université, Nancy, France; 2 L’Université Nantes Angers Le Mans, Institut National de la Santé et de la Recherche Médicale, U1063, Angers, France; 3 Université Pierre et Marie Curie Paris 06, Centre National de la Recherche Scientifique, Unité Mixte de Recherche 7190, Paris, France; 4 Institut National de la Santé et de la Recherche Médicale, U698, Paris Diderot University, Paris, France; I2MC INSERM UMR U1048, France

## Abstract

We aimed to characterize circulating microparticles in association with arterial stiffness, inflammation and endothelial dysfunction in aldosterone-salt-induced hypertension in rats and to investigate the preventive effects of red wine polyphenols. Uninephrectomized male Sprague-Dawley rats were treated with aldosterone-salt (1 µg.h^−1^), with or without administration of either red wine polyphenols, Provinols™ (20 mg.kg^−1^.day^−1^), or spironolactone (30 mg.kg^−1^.day^−1^) for 4 weeks. Microparticles, arterial stiffness, nitric oxide (NO) spin trapping, and mesenteric arterial function were measured. Aldosterone-salt rats showed increased microparticle levels, including those originating from platelets, endothelium and erythrocytes. Hypertension resulted in enhanced aortic stiffness accompanied by increased circulating and aortic NO levels and an upregulation of aortic inducible NO-synthase, NFκB, superoxide anions and nitrotyrosine. Flow-induced dilatation was reduced in mesenteric arteries. These effects were prevented by spironolactone. Provinols™ did not reduce arterial stiffness or systolic hypertension but had effects similar to those of spironolactone on endothelial function assessed by flow-mediated vasodilatation, microparticle generation, aortic NO levels and oxidative stress and apoptosis in the vessel wall. Neither the contractile response nor endothelium-dependent relaxation in mesenteric arteries differed between groups. The *in vivo* effects of Provinols™ were not mediated by mineralocorticoid receptors or changes in shear stress. In conclusion, vascular remodelling and endothelial dysfunction in aldosterone-salt-mediated hypertension are associated with increased circulating microparticles. Polyphenols prevent the enhanced release of microparticles, macrovascular inflammation and oxidative stress, and microvascular endothelial dysfunction independently of blood pressure, shear stress and mineralocorticoid receptor activation in a model of hyperaldosteronism.

## Introduction

Mineralocorticoid administration to uninephrectomized rats drinking a concentrated salt solution is a well known model of experimental hypertension and the degree of hypertension is dependent on the duration of the aldosterone-salt regimen. An increase in systolic arterial pressure (SAP) was observed after administration of aldosterone and salt for 4 weeks in rats. Key aspects of this model are the maintenance of the deleterious effect of salt in presence of relatively high levels of aldosterone and the presence of proteinuria and increased oxidative stress compared with uninephrectomized rats on a normal salt diet [1]. Furthermore, an increase in arterial stiffness, assessed by the incremental elastic modulus (Einc), was also observed within this time-frame [2]. The mineralocorticoid receptor antagonists, eplerenone and spironolactone, have been shown to prevent these arterial changes, together with the associated cardiac fibrosis, independently of left ventricular hypertrophy, via blood pressure reduction [3]. In hypertensive patients, eplerenone treatment has been recently shown to reduce stiffness in resistance arteries [4]. For endothelial function, a number of studies have shown that chronic treatment with aldosterone resulted in impaired acetylcholine-induced relaxation of the aorta in both normotensive and hypertensive rats resulting from inflammatory and oxidative processes [5].

Microparticles may also play a key role in endothelial dysfunction and vascular oxidative stress. Indeed, microparticles are small membrane vesicles that are shed from cells in response to activation and apoptosis in various disease states [6]. Because of their presence in the bloodstream, circulating microparticles may be considered to play a major role in interactions with circulating cells or the components of the vessel wall. Recently, it has been reported that hypertensive patients displayed both elevated levels of circulating endothelial microparticles and increased arterial stiffness assessed by brachial–ankle pulse wave velocity [7]. Furthermore, microparticles promote endothelial dysfunction in metabolic syndrome patients with moderate hypertension [8]. To the best of our knowledge, the circulating levels of microparticles according to their cellular origin have never been characterized in aldosterone-salt hypertension.

**Figure 1:**
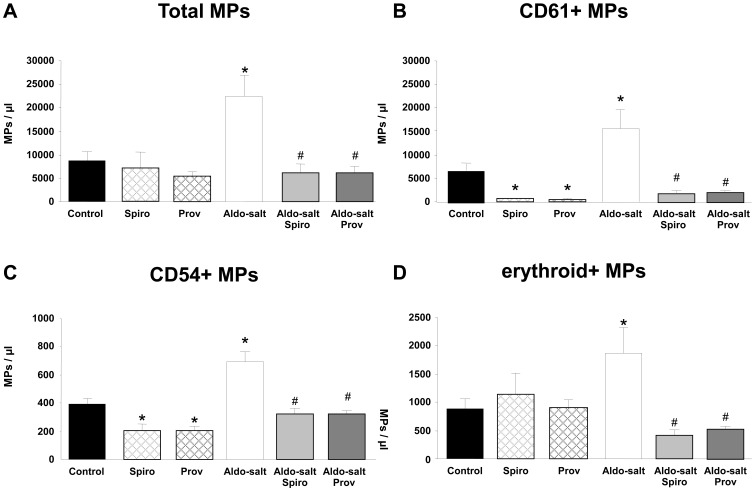
Circulating microparticles (MPs). Total MPs (A), platelet (CD61^+^)- (B), endothelial (CD54^+^)- (C) and erythrocyte- (D) derived MPs from control, spironolactone (Spiro), Provinols™ (Prov), aldosterone-salt (Aldo-salt), Aldo-salt Spiro and Aldo-salt Prov (n = 4–5 for each group). Values are means ± SEM, *P<0.05 *vs* control rats. ^#^ *P*<0.05 ^##^ *P*<0.01 *vs* Aldo-salt rats.

Red wine polyphenols, including Provinols™, possess beneficial properties for preventing cardiovascular disorders by their influence on nitric oxide (NO) balance and prevention of oxidative stress. These compounds can decrease blood pressure in normotensive rats [9], an effect which involves the NO pathway because polyphenols are able to induce *ex vivo* endothelium-dependent relaxation as a result of enhanced NO synthesis via endothelial NO synthase (eNOS) [10]. We [11], [12] and other groups [13] have provided evidence that Provinols™ reduce blood pressure in several experimental models of hypertension, and can correct cardiovascular remodelling and vascular dysfunction. Furthermore, our recent manuscript shows that delphinidin in Provinols™ activated the NO pathway (Src, ERK 1/2, eNOS, caveolin-1), leading to NO production in endothelial cells via activation of the α isoform of the oestrogen receptor [14]. An increase in NOS activity, the prevention of oxidative stress, and a reduction in the inflammatory process may explain the effects of Provinols™.

The uninephrectomized aldosterone-salt rat model is widely used for measuring the cardiovascular effects of aldosterone. We chose to use the classical reference group of uninephrectomized rats as controls and not uninephrectomized animals receiving a high salt diet, since the renin level is markedly decreased in these latter animals [15]. We consider also the model of aldosterone infusion and salt without uninephrectomy as an unsuitable control, because the specific involvement of mineralocorticoid receptor has not been definitively established. Indeed, the effects of mineralocorticoid receptor antagonism have never been described in this model [16].

**Table 1:** Effects of treatment by spironolactone (Spiro) or Provinols™ (Prov) on blood pressure, carotid diameter and organ weights in aldosterone-salt (Aldo-salt) treated rats.

Group	Control	Aldo-salt	Aldo-salt Spiro	Aldo-salt Prov
**N**	17	14	10	16
**Body weight (g)**	404±6	394±6	431±8* #	417±6#
**DAP (mmHg)**	124±5	135±6	134±3	141±5
**SAP (mmHg)**	167±6	192±8*	180±4	198±8*
**MAP (mmHg)**	138±5	154±7	149±3	160±6
**PP (mmHg)**	42±2	57±3*	46±1#	57±3*
**Diameter at MAP (mm)**	1.26±0.04	1.32±0.05	1.22±0.07	1.25±0.05
**Heart weight (g)**	1.31±0.05	1.64±0.05*	1.53±0.05* #	1.60±0.05*
**Kidney weight (g)**	2.02±0.04	2.44±0.06*	2.48±0.06*	2.60±0.10*

DAP: diastolic arterial pressure; SAP: systolic arterial pressure; MAP: mean arterial blood pressure; PP: pulse pressure.

Values are means ± SEM.

* *P*<0.05 *vs* Control.

# *P*<0.05 *vs* Aldo-salt.

In the present study, our aim was first to characterize circulating microparticles according to their cellular origin and to study their role in association with arterial stiffness, vascular inflammation and endothelial dysfunction in aldosterone-salt-induced hypertension from uninephrectomized rats. Secondly, we investigated the cardio-protective effects of Provinols™ on the consequences of aldosterone-salt-induced hypertension on the above parameters, i.e. microparticles, large artery stiffness, inflammation and oxidative stress, and flow-induced dilatation in uninephrectomized rats. The effects of Provinols™ alone were compared with those of spironolactone alone in another series of uninephrectomized control rats.

**Figure 2:**
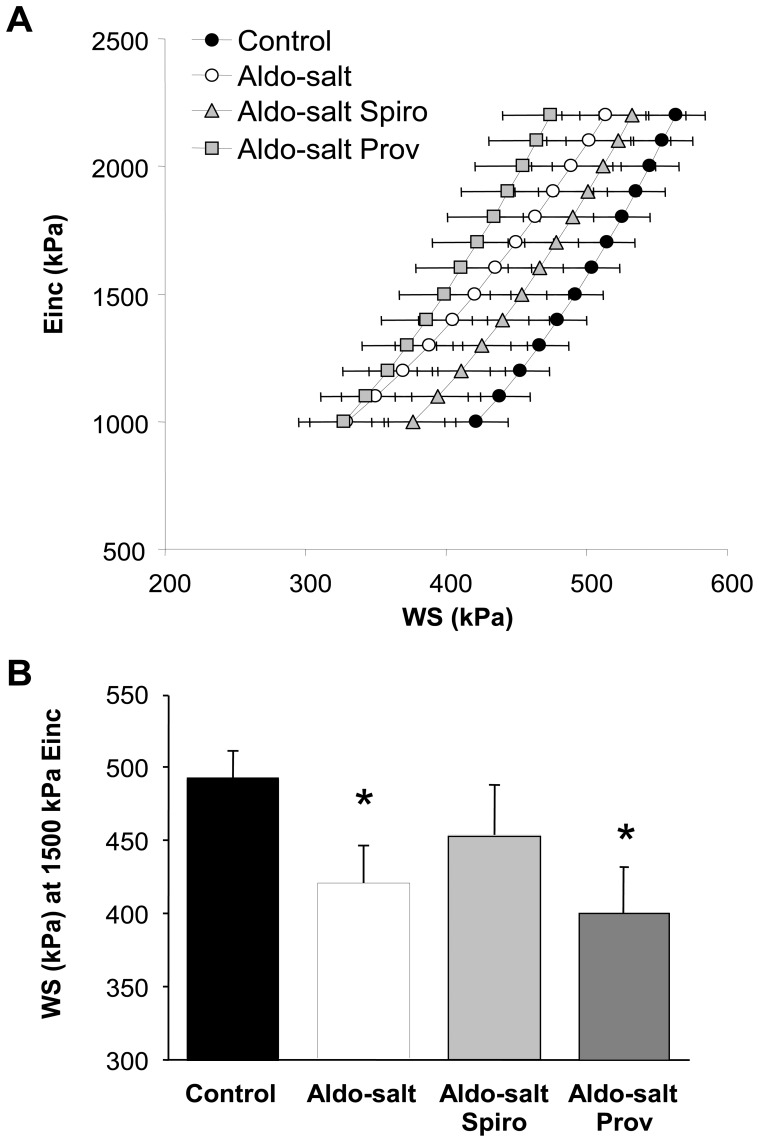
Carotid artery incremental elastic modulus-wall stress (Einc-WS) curves. **A.** Einc-WS curves from Control, aldosterone-salt (Aldo-salt), Aldo-salt spironolactone (Aldo-salt Spiro) and Aldo-salt Provinols™ (Aldo-salt Prov) rats (n = 5 for each group). **B.** Mean value of WS at 1500 kPa of Einc. Values are means ± SEM, *P<0.05 *vs* control rats.

## Methods

### Animals

Male Sprague-Dawley (SD) rats (n = 149), uninephrectomized at 7 weeks of age, were purchased when 8 weeks old from Charles River (France). All rats were housed in individual cages and were fed a standard rat chow and tap water *ad libitum*. They were maintained in a quiet room at constant temperature (20 to 22°C) and humidity (50 to 60%). The rats were randomly divided into 6 groups. In the control group (n = 41), the uninephrectomized SD rats received normal food for 4 weeks without aldosterone (Sigma-Aldrich, St Louis, MO) administration. In the spironolactone (Sigma-Aldrich) group (n = 12), rats received spironolactone (30 mg.kg^−1^.day^−1^) in the food (based on an average consumption of 25 g.day^−1^) for 4 weeks without aldosterone administration. In the Provinols™ group (n = 12), rats received Provinols™ 20 mg.kg^−1^.day^−1^ in their food [17] without aldosterone administration. In the aldosterone-salt group (n = 28), osmotic mini-pumps were implanted subcutaneously between the shoulder blades (Alzet model 2004, 1 µg.h^−1^) for continuous subcutaneous infusion for 4 weeks. Osmotic mini-pumps were filled with aldosterone and incubated for several hours at 37°C prior to implantation. Surgery was performed using an aseptic technique and the rats were anaesthetized with isofluorane 1.5% in oxygen. The aldosterone-salt group rats received 1% NaCl solution (plus 0.1% KCl) to drink, provided in a single drinking burette. In the aldosterone-salt spironolactone group (n = 26), aldosterone-salt rats received spironolactone (30 mg.kg^−1^.day^−1^) in the food and in the aldosterone-salt Provinols™ group (n = 30), aldosterone-salt rats received Provinols™ (20 mg.kg^−1^.day^−1^) in their food. Provinols™ were obtained from the Société Française des Distilleries Union de Coopératives Agricoles (Vallon Pont d'Arc, France). The composition, in mg.g^−1^ of dry powder, is: 480 proanthocyanidins, 61 total anthocyanins, 19 free anthocyanins, 38 catechin, 18 hydroxycinnamic acids, 14 flavonols and 370 polymeric tannins. All procedures were conducted in accordance with the Guide for the Care and Use of Laboratory Animals published by the US National Institute of Health and were approved by the local ethical committee “Comité regional Nancy-Lorraine/Nord-Est” (n° B54-547-20).

**Table 2:** Effects of treatment by Spironolactone (Spiro) or Provinols™ (Prov) on aortic medial composition in aldosterone-salt (Aldo-salt) treated rats.

Group	Control	Aldo-salt	Aldo-salt-Spiro	Aldo-salt-Prov
**N**	11	9	10	10
**Dry weight (mg/cm)**	2.69±0.07	3.03±0.15*	2.90±0.08	2.93±0.09
**Cell proteins (% dry wt)**	13.3±0.3	16.5±0.4*	14.4±0.6^#^	16.8±0.5*
**Cell proteins (mg/cm)**	0.34±0.01	0.48±0.03*	0.40±0.02*^#^	0.47±0.01*
**N**	5	4	4	6
**Elastin (% dry wt)**	52.7±0.3	43.2±1.2*	48.5±1.0*^#^	46.6±0.8*^#^
**Elastin (mg/cm)**	1.39±0.06	1.32±0.07	1.42±0.04	1.24±0.05
**Collagen (% dry wt)**	13.6±0.2	12.8±0.2	12.9±0.7	13.0±0.3
**Collagen (mg/cm)**	0.36±0.02	0.39±0.030	0.38±0.03	0.35±0.02
**Elastin/collagen**	3.89±0.07	3.37±0.09*	3.80±0.20	3.60±0.10

Values are means ± SEM *P*<0.05 *vs* Control, ^#^ *P*<0.05 *vs* Aldo-salt.

### Microparticle Isolation and Characterization

Blood obtained by intracardiac puncture was collected in citrated tubes and processed for assay within 2 hours. Samples were centrifuged 3 minutes at 1900 *g* and then 4 minutes at 5000 *g* to obtain platelet-free plasma (PFP) and stored at −80°C until subsequent use. Discrimination between the various membrane microparticle subpopulations present in PFP was achieved by analyzing the expression of membrane-specific antigens, as previously described [18]. Quantification and phenotype of endothelial, platelet, and erythrocyte microparticles were performed using anti-CD54-biotin (1.5 µg.ml^−1^), anti-CD61-FITC (2.5 µg.ml^−1^) (BioLegend, San Diego, CA) and anti-erythroid cell-biotin (2.5 µg.ml^−1^) (BD Biosciences, San Jose, CA) labeling. For biotin-conjugated antibodies, streptavidin-FITC (2.5 µg.ml^−1^) (Sigma-Aldrich) was added for 15 minutes. Eight µl of plasma were incubated with 5 µl of specific antibody (Beckman Coulter, Villepinte, France). Irrelevant rat IgG was used as an isotype-matched negative control for each sample. After 45 minutes of incubation at room temperature, samples were diluted in 300 µl of 0.9% saline salt solution. Then 8 µl of Flowcount beads were added and samples were analyzed in a flow cytometer 500 MPL system (Beckman Coulter).

**Figure 3:**
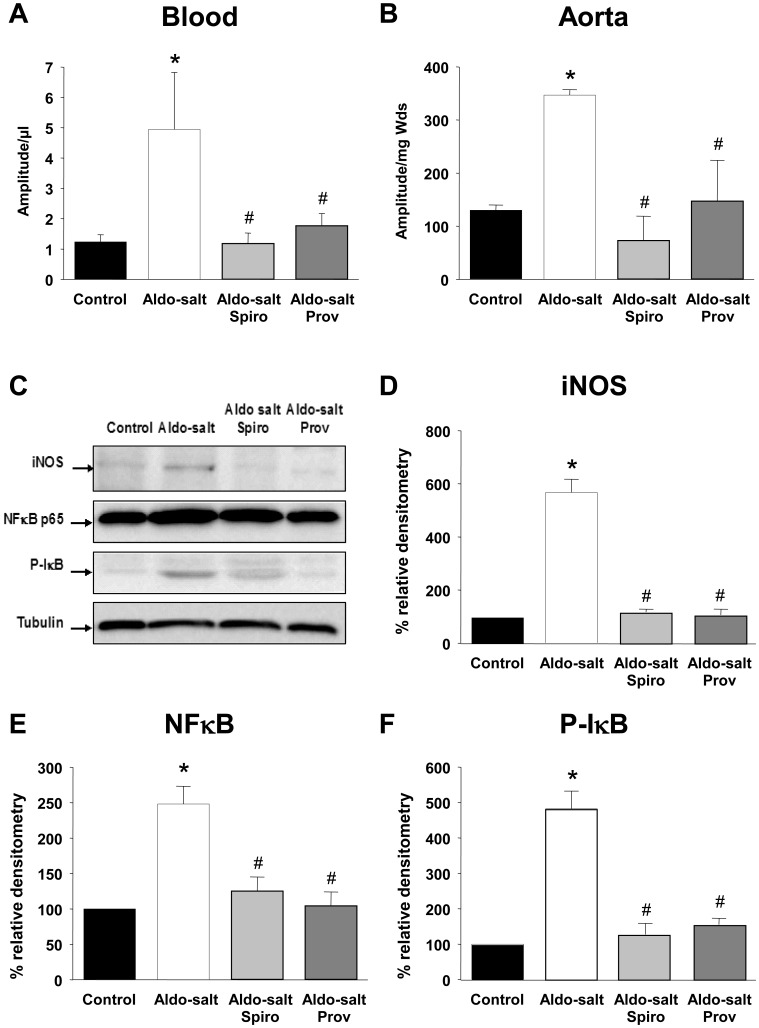
NO production, iNOS and NFκB pathways. Quantification of the amplitude of NO-Fe(DETC)_2_ signal per µl in blood (A) and per mg of dried sample in aorta (B). Values are means ± SEM, *P<0.05 *vs* control rats, #P<0.05 *vs* Aldo-salt rats (n = 4–5 for each group). Western blot (C) and densitometric analysis revealing iNOS (D), NFκB p65 (E) expression and phosphorylated I-κB alpha (P-IκB; F) in aortae. Values are means ± SEM, *P<0.05 *vs* control rats, ^#^ *P*<0.05 *vs* Aldo-salt rats.

### Von Willebrand Factor ELISA

Circulating von Willebrand factor (vWF) antigen was measured by ELISA (Asserachrom®, Diagnostica Stago, Asnières, France). Each plasma sample was assayed at two different dilutions chosen in order to interpolate results using the calibration curve obtained with the human calibrator supplied within the kit.

### Carotid Artery Stiffness

We performed simultaneous recording of arterial diameter (left carotid artery) and blood pressure (right carotid artery) in pentobarbital-anesthetized rats as has been described previously [2]. Arterial diameter measurement was obtained by using an ultrasonic echo-tracking device (Diarad-00, Asulab SA). The relationship between the pressure (P) and the lumen cross-sectional area (LCSA) was fitted with the model of Tardy et al. [19] by using an arctangent function and three optimal fit parameters (α, β, γ) as follows:
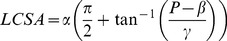
(1)


Carotid cross-sectional distensibility (Dist), a derivative of this function, was used to assess the global elastic behaviour of the artery. Circumferential wall stress (σ) and Einc, which characterises the intrinsic mechanical properties of the wall material, were calculated with the above-mentioned parameters. Dist, σ and Einc are given by the following equations:
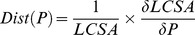
(2)

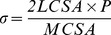
(3)

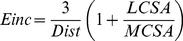
(4)where the media cross sectional area (MCSA) was determined by histomorphometry. The reproducibility was 3±1% (inter-observer coefficient of variation of carotid diameter).

### Aortic Composition

Insoluble elastin, total collagen and cell protein contents were measured on descending thoracic aortae without homogenization, as described previously [20]. Briefly, aortic segments were opened longitudinally, the media separated from the adventitia and the medial length measured under a microscope. Media were then defatted, dried and their dry weight recorded. Medial cell proteins were extracted by 0.3% sodium dodecyl sulfate (SDS) and subsequently assayed and insoluble elastin was purified by the hot alkali method and quantified by weighing. Proteins in the NaOH extract were then hydrolysed, and total medial collagen was quantified by assaying hydroxyproline in the hydrolysate, using a colorimetric assay.

### NO Spin Trapping and Electronic Paramagnetic Resonance Technique (EPR) Studies

The detection of NO production was performed using the technique with Fe^2+^ diethyldithio-carbamate (DETC, Sigma-Aldrich) as spin trap [21]. To measure NO production in blood, we injected 400 mg.kg^−1^ of DETC (1 µl.g^−1^ weight) intraperitoneally and 40 mg.kg^−1^ of FeSO_4_.7H_2_O (1 µl.g^−1^ weight), in a solution containing sodium citrate dehydrate 200 mg.ml^−1^, subcutaneously. After 30 minutes, rats were anesthetized with isoflurane and killed, and venous blood was obtained for NO measurements. These studies were performed on a table-top x-band spectrometer Miniscope (Magnettech, Berlin, Germany). Recordings were made at 77°K, using a Dewar flask. Instrument settings were 10 mW of microwave power, 1 mT of amplitude modulation, 100 kHz of modulation frequency, 60 seconds of sweep time and 3 scans. Blood samples exhibited an EPR characteristic of signals derived from NO-Fe(DETC)_2_ [22]. The quantitative measurement of the NO-Fe(DETC)_2_ signal amplitude was expressed in relative units.µl^−1^ of blood (amplitude per µl).

In another set of experiments, animals were killed and aorta dissected and incubated for NO production in 250 µl of Krebs–Hepes buffer solution, then treated with 250 µl of Fe(DETC)_2_ and incubated for 45 minutes at 37°C. NO detection was measured in situ by EPR as described above, and values were corrected for the dry weight of the sample (dehydrated sample) in mg and expressed as amplitude signal per mg (W_ds_).

**Figure 4:**
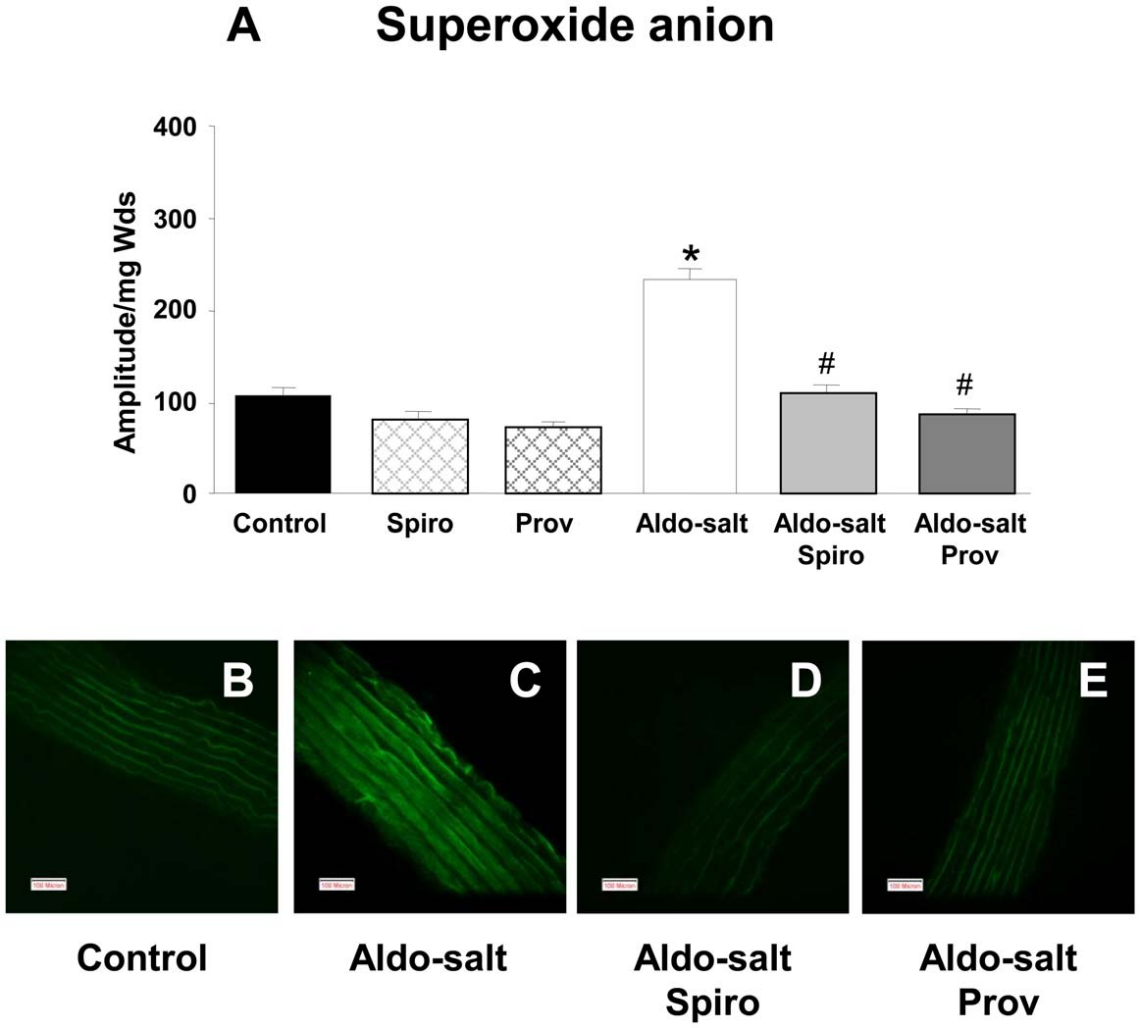
Superoxide anion and nitrotyrosine staining in aorta. Quantification of the amplitude of O_2_^–^CMH signal (A) and immunohistochemical staining for nitrotyrosine (B-E) in aortae (n = 4–5 for each group). Values are means ± SEM, *P<0.05 *vs* control rats, ^#^ *P*<0.05 *vs* Aldo-salt rats.

### Superoxide Anion (O_2_^−^) Determination by EPR

Aorta was dissected and allowed to equilibrate in deferoxamin-chelated Krebs-Hepes solution containing 1-hydroxy-3-methoxycarbonyl-2,2,5,5-tetramethylpyrrolidin (CMH; Noxygen, Mainz, Germany) (500 µM), deferoxamine (25 µM) and DETC (5 µM) at 37°C for 20 minutes. O_2_^−^ detection was measured in situ by EPR, and values are expressed as amplitude of signal per mg dehydrated tissue (dry weight).

### Western Blotting

Seventy-five µg of total proteins from aortae and 20 µg of total proteins from VSMCs were loaded onto 8% or 10% SDS-PAGE. Proteins were transferred to nitrocellulose membranes and incubated overnight (4°C) with a monoclonal mouse anti-iNOS (1∶500, BD Transduction Laboratories), a mouse anti-IκBalpha phosphorylated (Ser32/36) (1∶1000, USBiological, Swampscott, MA), a rabbit polyclonal NFκB p65 antibody (1∶400, Abcam, Cambridge, UK), anti-caspase-8 and anti-caspase-3 (1∶1000, Cell Signaling Technology, Danvers, MA) or a mouse tubulin antibody (1∶200, Sigma-Aldrich). Bound antibodies were detected with a secondary peroxidase-conjugated anti-mouse or anti-rabbit IgG (1∶500, Pierce, Perbio Science France, Brebières, France). The bands were visualized using the enhanced chemiluminescence system (Super Signal West Femto Maximum Sensitivity Substrate, Pierce), quantified by densitometry and normalized to tubulin or β-actin expression. Results were expressed as the percentage of staining compared with control aortae or VSMCs taken as 100%.

### Aortic Nitrotyrosine Staining

Fixed sections from the aortae were incubated in blocking buffer (5% non-fat dry milk) for nitrotyrosine. Then, tissue sections were incubated overnight (4°C) with a mouse monoclonal anti-nitrotyrosine (clone 1A6) antibody (1∶100, Millipore, Billerica, MA, ref: 05-233) in blocking buffer. Three washes were followed by incubation with secondary murine fluorescence-labeled antibody Alexa fluor-488 (1∶100, Molecular Probes, Cergy Pontoise, France). Controls were performed by omission of the first or second antibody. After washing, vessel sections were mounted on glass slides. Confocal equipment mounted on a Nikon Eclipse, TE 200-S, inverted microscope was used for optical sectioning of the tissue. Digital image recording was performed using the QED In Vivo Software.

**Figure 5:**
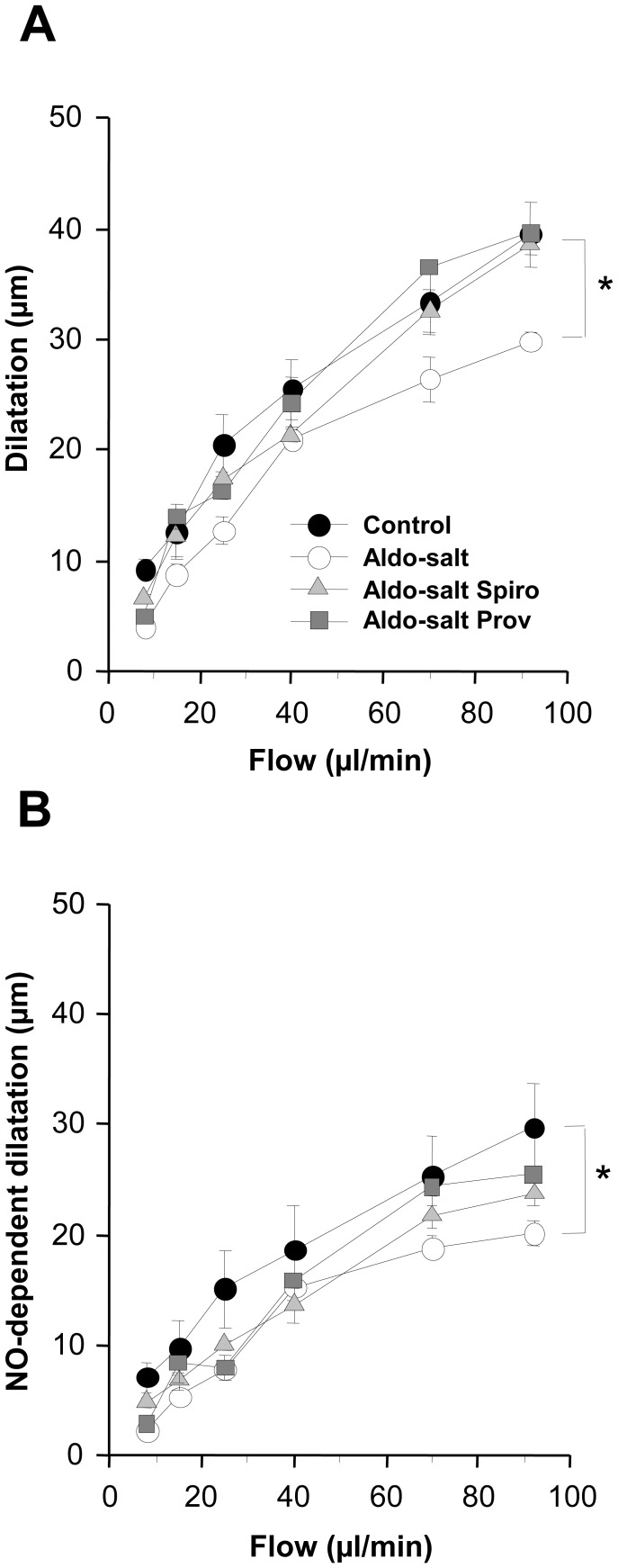
Endothelial function in small mesenteric arteries. A: Flow-induced dilatation in small mesenteric arteries from control, aldosterone-salt (Aldo-salt), aldosterone-salt spironolactone (Aldo-salt Spiro) and aldosterone-salt Provinols™ (Aldo-salt Prov) calculated as Δdiameter. Absolute values for baseline diameters were 133±9, 136±7, 142±5 and 153±6 µm, respectively. *P<0.05 Aldo-salt *vs* control. B: NO-dependent dilatation calculated as the difference between the dilatation in basal conditions and the dilatation in the presence of 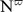 nitro-L-arginine (L-NA, 100 µM). Absolute values for baseline diameters ere 133±4, 135±4, 143±6 and 154±4 µm, respectively. n = 5 for each group. Values are means ± SEM. *P<0.05 Aldo-salt *vs* control vessels.

### Vascular Reactivity

Second generation branches of small mesenteric arteries (SMA) were carefully removed and arterial segments were mounted on a wire myograph filled with physiological salt solution (PSS) of the following composition in mM: NaCl 130, NaHCO_3_ 14.9, KCl 3.7, KH_2_PO_4_ 1.2, MgSO_4_.7H_2_O 1.2, CaCl_2_.H_2_O 1.6 and glucose 11, under normalized tension, as previously described [10]. Briefly, SMA were stretched to 200 mg, lengths that yielded circumferences equivalent to 90% of those which the vessels would have had with an intramural pressure of 100 mmHg (i.e., the pressure corresponds to the physiological value). PSS was kept at 37°C and gassed with 95% O_2_ and 5% CO_2_ at pH 7.4. Mechanical activity was recorded isometrically by a force transducer (Danish Myo Technology, Aarhus, Denmark). After setting the vessels to their working length, the contractile response to KCl (80 mM) was assessed. After washing of arteries, challenges with phenylephrine (Sigma Aldrich) were performed to elicit a contractile response and to test their maximal contractile capacity. Arteries were pre-contracted at 80% of their maximal contraction with phenylephrine. When the contraction reached a plateau, cumulative application of acetylcholine (1 nM to 10 µM, Sigma Aldrich) was performed. The presence of functional endothelium was assessed by the ability to induce more than 50% relaxation of vessels pre-contracted with phenylephrine. Parameters of vascular sensitivity were analyzed by means of pD_2_ =  -log EC_50_, EC_50_ being the molar concentration of the agonist that produces 50% of the maximal effect; EC_50_ values were calculated by logit-log regression.

#### Flow-mediated dilatation

Isolated SMAs (∼130–160 µm of diameter) were cannulated at both ends in a video-monitored perfusion system (Living Systems Instrumentation, Burlington, VT). Arteries were bathed in physiological salt solution at pH 7.4, PO_2_ 160 mmHg, PCO_2_ 37 mmHg, as described previously [23]. The presence of endothelium was tested by applying acetylcholine after pre-contraction of the arteries with KCl-depolarization (80 mM). Diameter changes were measured when intra-luminal pressure was increased from 10 to 125 mmHg. Pressure was then set at 75 mmHg and dilatation in response to flow was evaluated. Dilatation response-to-flow experiments were repeated in series in presence of the NOS inhibitor, 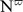 nitro-L-arginine (L-NA, 100 µM, Sigma-Aldrich). The NO-dependent dilatation was calculated as the difference between the dilatation without inhibitor and that in the presence of L-NA.

### Shear Stress-induced Microparticle Formation

Shear-induced blood cell activation was evaluated *in vitro* using an erythroaggregometer (Couette flow, SD Medical), subjecting citrated anticoagulated whole blood (from Sprague Dawley rats via a carotid catheter under isoflourane anaesthesia) (129 mM; v/v 9∶1) to shear rates of 300 s^−1^ for 2 minutes at 37°C. Samples were incubated with aldosterone at 10 nM for 10 min. The following antagonists were added 10 min prior to the addition of aldosterone: the mineralcorticoid receptor antagonist, spironolactone, at 1 µM, Provinols™ at 0.01 g.l^−1^ or the oestrogen receptor antagonist, fulvestrant (Fulv; Sigma-Aldrich), at 1 µM. Controls were performed in which citrated whole blood was in contact with the cup and bob for 2 minutes at 37°C but not exposed to shear stress. Samples were then centrifuged for 10 minutes at 190 *g* to obtain platelet-rich plasma (PRP) and then for 3 minutes at 1900 *g*. The supernatant was centrifuged for 4 min at 5000 *g* to obtain the platelet-poor plasma (PPP). Platelet activation and the generation of cell-derived microparticles in sheared blood samples were evaluated using the chromogenic assay measuring the phospholipid-related procoagulant activity (PPA) as previously described [24].

**Figure 6:**
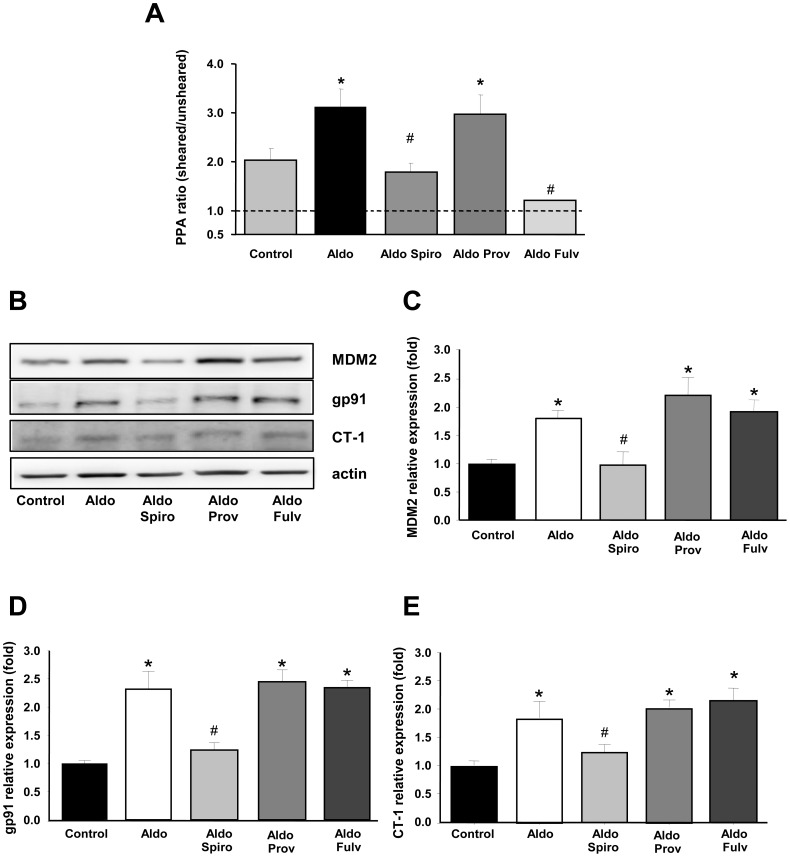
Shear stress and microparticle formation. (A) Phospholipid-related procoagulant activity (PPA) values are expressed as a ratio between sheared and unsheared data from blood samples treated with aldosterone (Aldo), aldosterone plus spironolactone (Aldo+Spiro) and aldosterone plus Provinols™ (Aldo+Prov) groups (n = 4–5 for each group). Values are means ± SEM, *P<0.05 vs control (PBS). #P<0.05 vs aldosterone-treated samples. (B-E). Mineralocorticoid receptor antagonist activity. VSMCs were preincubated with spironolactone (Spiro), Provinols™ (Prov) or fulvestrant (Fluv) for 1 hour, and then aldosterone (Aldo) was added for 24 hours for protein analysis. Changes in MDM2 (C), gp91 (D) and CT-1 (E) protein expression were assayed by Western Blotting and normalized with β-actin in triplicate. A representative Western blot (B) and the histograms with bars represent the means ± SEM of triplicates in three independent experiments. *P<0.01 vs Control.

### Vascular Smooth Muscle Cell Isolation and Culture

Primary rat aortic vascular smooth muscle cells (VSMCs) were isolated by using standard enzymatic dissociation techniques, as previously reported [25]. Cells were plated and grown in Dulbecco’s modified Eagle’s medium (DMEM) supplemented with 5% fetal bovine serum, and cultured at 37°C in air containing 5% CO_2_. Cells were used at passage 2. Before treatment, cells were serum-starved for 12 hours.

MDM2, cardiotrophin-1 (CT-1) and gp91 are genes induced by aldosterone in a mineralocorticoid-dependent pathway [25]–[27]. VSMCs were incubated with aldosterone at 10 nM for 24 hours. The following antagonists were added one hour prior to the addition of aldosterone: the mineral corticoid receptor antagonist, spironolactone, at 1 µM, Provinols™ at 0.01 g.L^−1^, or the oestrogen receptor antagonist, fulvestrant, at 1 µM.

### Statistical Analysis

All values are expressed as means ± SEM. One-way analysis of variance was performed to compare the different groups of rats. The Fisher test was used for inter-group comparisons. Einc was log transformed to generate linear relationships [28]. After this transformation, we calculated the mean slopes of the curves. If the slopes were not significantly different, we compared the curves within the common range of Einc for WS by calculating WS at 1500 kPa of Einc. Differences were considered significant at values of *P*<0.05. Statistical analyses of PD_2_ values and maximal effects of acetylcholine were performed by two-way analysis of variance, and nonparametric Mann-Whitney U tests or analysis of variance for repeated measures and subsequent Bonferroni post hoc test.

**Figure 7:**
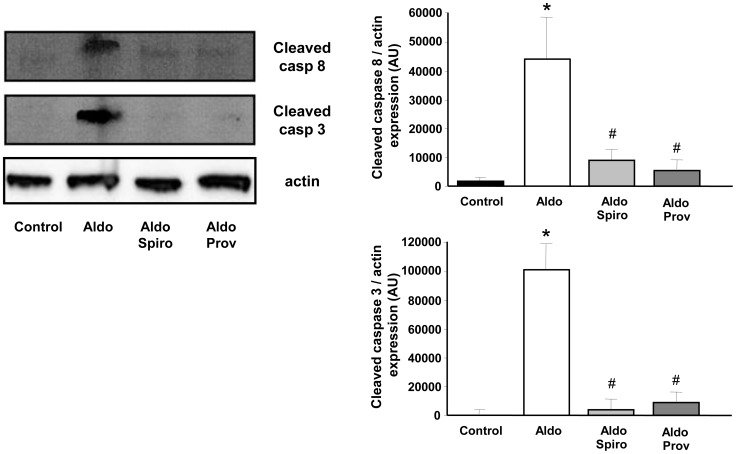
Effects on vessel wall apoptosis. Western blot and densitometric analysis revealing expression of cleaved caspase-8 and caspase-3 in aortae. Data are representative of at least four independent experiments. Values are means ± SEM, *P<0.05 *vs* control rats, ^#^ *P*<0.05 *vs* Aldo-salt rats.

## Results

### Arterial Pressure and Organ Weights

Aldosterone-salt treatment increased significantly SAP and pulse pressure (PP) compared to controls. There were no significant changes observed in body weight, diastolic or mean blood pressures or in carotid artery diameter. The significant increase in SAP and PP due to aldosterone-salt treatment was prevented by spironolactone treatment but not by Provinols™ (Table 1). When administered alone, spironolactone but not Provinols™ exerted an inhibitory effect on blood pressure (Table S1). Spironolactone and Provinols™ treatment increased body weight compared to the aldosterone-salt group (Table 1). The increases in heart and kidney weights induced by the aldosterone-salt treatment were not influenced by Provinols™ whereas spironolactone partially prevented the increase in heart weight.

### Microparticle Counts and their Cellular Origin

Aldosterone-salt treatment increased significantly the total number of circulating microparticles when compared to control (Figure 1A). This increase in the total number of microparticles was completely prevented by both spironolactone and Provinols™. Neither spironolactone nor Provinols™ alone had any effect on the total number of microparticles (Figure 1A). Phenotypical characterization of the cellular origin of microparticles showed significant increases in platelet-, endothelial- and erythrocyte-derived microparticles in aldosterone-salt-treated rats (Figures 1B–D) which were all inhibited by both spironolactone and Provinols™. In addition, both spironolactone and Provinols™ alone significantly reduced platelet- and endothelial-derived microparticles, without modifying erythrocyte-derived microparticle number (Figure 1B–D).

### Von Willebrand Factor

There was an increase in circulating vWF in aldosterone-salt rats (3.4±0.4 versus 2.2±0.3 µg.ml^−1^ in controls; n = 7 and 11 respectively; p = 0.02) which was inhibited by both spironolactone (2.2±0.3 µg.ml^−1^; n = 9) and Provinols™ (2.1±0.3 µg.ml^−1^; n = 10).

### Incremental Elastic Modulus-wall Stress Curves

Figure 2A shows Einc-WS curves in four groups of rats. To compare these curves, we calculated the mean WS at 1500 kPa of Einc (Figure 2B). Comparison of Einc-WS curves from aldosterone-salt and control rats showed a significant reduction in WS at 1500 kPa of Einc in aldosterone-salt rats indicating increased arterial stiffness. In the spironolactone group, this increase was not observed. However, Provinols™ did not significantly prevent aldosterone-salt-induced arterial stiffness. The Einc-WS curves from rats treated with spironolactone or Provinols™ alone were not different from the control group (Figure S1).

### Aortic Composition

Thoracic aortic medial dry weight per cm length, and cell protein content were increased significantly by the aldosterone-salt treatment compared with control rats (Table 2), indicating that hypertrophy of the media had occurred. The increased dry weight reflects not only the increase in smooth muscle cell content but also an increase in some extracellular components not quantified here (principally proteoglycans and glycoproteins) and deduced by substraction. The significant increase in aortic dry weight due to aldosterone-salt was not observed under co-treatment with spironolactone or Provinols™. However, only spironolactone, but not Provinols™, was able to significantly attenuate the aldosterone-salt-induced increase in cell proteins.

A significant decrease in the percentage of elastin in the thoracic aorta with aldosterone-salt treatment was observed, which was only partially corrected by both spironolactone and Provinols™ treatments. There were no changes in collagen with aldosterone-salt treatment. However, as the proportion of elastin was decreased, there was a significant reduction in the elastin to collagen ratio after aldosterone-salt treatment. Both spironolactone and Provinols™ partially prevented this reduction in elastin/collagen providing values approaching those of controls.

### NO production, iNOS, NFκB p65 and phosphorylated-IκB alpha activation

The NO-Fe(DETC)_2_ EPR signal was greater in blood and aorta from aldosterone-salt treated rats than in control rats (Figures 3A and 3B, respectively). This increase was completely prevented by spironolactone or Provinols™. Neither spironolactone nor Provinols™ alone had any effect on aortic NO production (Figure S2).

Western blot analysis showed an increase in iNOS expression in aortae from aldosterone-salt rats compared to those from control rats (Figures 3C and 3D). Much less staining was observed following treatment with spironolactone or Provinols™. The iNOS expression was associated with an increase in NFκB p65 expression in aortae from aldosterone-salt rats compared to control rats (Figures 3C and 3E). Activation of the transcriptional factor, NFκB p65, in aortae from aldosterone-salt treated rats was confirmed by the increased levels of phosphorylated-IκB alpha compared to control vessels (Figures 3C and 3F). All these effects of aldosterone-salt treatment on iNOS and NFκB p65 expression and phosphorylated-IκB alpha were completely prevented both by spironolactone and by Provinols™.

### Oxidative and Nitrosative Stresses

EPR measurement of oxidative stress demonstrated that aortae from aldosterone-salt-treated rats displayed an increased O_2_^-^ production compared with controls that was prevented by both spironolactone and Provinols™ (Figure 4A). Neither spironolactone alone nor Provinols™ alone affected O_2_^-^ production (Figure 4).

Aortae from aldosterone-salt rats also showed a marked increase in nitrotyrosine staining, reflecting an increased degree of protein nitration in the vascular wall (Figures 4B and C). Weak staining, comparable to that obtained in control aorta, was found in vessels from aldosterone-salt rats treated either with spironolactone or Provinols™ (Figures 4D and E).

**Table 3:** Contractile effects of KCl (80 mM) and endothelium-dependent relaxation to acetylcholine in rat mesenteric resistance arteries.

	KCl-induced contraction mN/mm	Endothelium-dependent relaxation to acetylcholine	
		pD_2_	Maximal effect (% relaxation)
**Control**	4.45±0.44	7.50±0.15	92.9±5.1
**Spiro**	3.76±0.17	7.06±0.13	92.2±2.4
**Prov**	3.71±0.68	7.13±0.22	87.1±5.8
**Aldo-salt**	4.91±0.46	7.37±0.06	98.3±1.3
**Aldo-salt Spiro**	3.92±0.48	7.41±0.17	97.8±1.2
**Aldo-salt Prov**	4.02±0.29	7.24±0.21	97.2±1.6

Spironolactone  =  Spiro; Provinols™  =  Prov; Aldosterone-salt  =  Aldo-salt.

Values are means ± SEM (n = 4–6).

* *P*<0.05 *vs* Spiro.

### Vascular Reactivity in Small Mesenteric Arteries

The contractile response to KCl-depolarization (80 mM) did not differ between control rats and the other groups. However, contraction to KCl was increased in aldosterone-salt rats when compared with the treatment spironolactone alone. Endothelium-dependent relaxation in response to acetylcholine was not significantly different in vessels harvested from the different groups of rats (Table 3).

Flow increases induced a significant vasodilator response in all the 4 groups of rats studied (Figure 5A). This flow-induced dilatation was significantly reduced in small mesenteric arteries from the aldosterone-salt-treated rats compared to those from control. Treatment by spironolactone and Provinols™ normalized the effect of aldosterone-salt on flow-induced dilatation.

Moreover, the NO-dependent dilatation (Figure 5B) was calculated as the difference between the dilatation without L-NA, represented in Figure 5A, and the dilatation in presence of L-NA (not shown). The NO-component of flow-induced dilatation was reduced in arteries from aldosterone-salt-treated rats compared to vessels from control rats. Treatment either with spironolactone or Provinols™ largely corrected this aldosterone-salt-induced effect.

### Shear Stress and Microparticle Formation

Figure 6A shows the PPA ratio values in PPP obtained from sheared and unsheared samples of total rat blood. Shear stress induced an increase in microparticle production in control samples. Treatment with aldosterone potentiated this shear-induced production. This effect was completely inhibited by spironolactone but not by Provinols™. This absence of mineralocorticoid receptor antagonist activity of Provinols™ was further supported by the inability of Provinols™ to block the aldosterone-induced increase in the expression of MDM2, gp91 and cardiotrophin-1 (CT-1) by cultured primary rat vascular smooth muscle cells (Figure 6 B–E). Estrogen receptor blockade with fulvestrant inhibited the effect of shear stress. However, in the absence of shear stress, aldosterone did not induce microparticle production by cultured human aortic endothelial cells (122±6 versus 130±4 nM equivalent phosphatidylserine in control cells).

### Spironolactone and Provinols Prevent Aldosterone-induced Apoptosis in the Vessel Wall

As shown in Figure 7, vessels from aldosterone-salt group display expression of cleaved caspase-8 and caspase-3, indicating that aldosterone-salt treatment induces apoptosis of vessel wall. In addition, both Spirolactone and Provinols prevented aldosterone-salt-induced apoptosis as reflected by the reduction of caspase cleavage.

## Discussion

We report that aldosterone-salt-induced hypertension produced a marked increase in both circulating microparticles, especially those from platelets, endothelium and erythrocytes, and circulating vWF. The microparticle increase was accompanied by increases in aortic stiffness, oxidative and nitrosative stresses and microvascular endothelial dysfunction as well as induction of apoptosis in the vessel wall. Of particular interest is that Provinols™ prevented changes in circulating microparticles, microvascular endothelial dysfunction, oxidative and nitrosative stress and apoptosis. The effects of these polyphenols were not mediated by mineralocorticoid receptors or changes in shear stress. However, Provinols™ did not block the aldosterone-salt-induced increases in aortic hypertrophy, stiffness or SAP.

Microparticle levels reflect the severity of endothelial alterations and of platelet activation. Although the precise mechanisms of microparticle production remain unclear, it has been emphasized that shear stress contributes to microparticle release whatever their cellular origin, as we have demonstrated in our *in vitro* studies. An effect of hypertension on microparticle production is supported by the correlation between plasma levels of endothelial and platelet microparticles and blood pressure in patients with severe untreated hypertension [29]. Besides reflecting the state of the parent cells, there is growing consensus on the many facets of microparticles which can act as diffusible messengers with either beneficial or deleterious missions [6], [30]. In particular, a functional or causative role of microparticles on endothelial dysfunction has been reported in several studies involving *in vitro* incubation of microparticles with endothelial cells or rings from thoracic aortae or their injection into mice [8], [31]–[36]. Although a possible direct effect of microparticles was not assessed on different types of arteries, they might contribute to the blood vessel inflammation observed in the present study (see below). For example, microparticles harvested from patients with pre-eclampsia induce activation of the transcription factor RelA/NFκB associated with an up-regulation of proinflammatory protein expression, namely iNOS and COX-2, in the vessel wall. The proinflammatory properties of these microparticles lead to the observation of surrogate signs of oxidative and nitrosative stress in the vascular wall [26]. The same type of inflammation was seen in the aorta from aldosterone-salt-treated rats. Finally, spironolactone or Provinols™ alone decreased endothelial-derived microparticle levels. Spironolactone [37] as well as red wine polyphenols [38] protect endothelial cells against apoptosis, a cellular process releasing microparticles. Similar mechanisms may explain the effects of spironolactone and Provinols™ on endothelial-derived microparticle release. This hypothesis is reinforced by the fact that spironolactone or Provinols™ prevented apoptosis induced by aldosterone-salt treatment at the level of the vessel wall.

Blockade of the aldosterone pathway by spironolactone or treatment by Provinols™ prevented the increase in circulating microparticles and plasma vWF as well as the associated vascular inflammation. Recently, aldosterone has been reported to activate endothelial vWF exocytosis at the cellular level [39]. Although microparticle generation and vWF release represent distinctive phenomena they may both be involved in aldosterone-salt-induced vascular inflammation. Even though it has been shown that selective mineralocorticoid receptor inhibition decreases platelet activation, the mechanism responsible for these effects has not been defined [40]. Lijnen and Petrov [41] described that spironolactone decreased intraerythrocyte and intraplatelet Na^+^ and Ca^2+^ by decreasing Na^+^-pump activity. It is possible that the reduction in platelet microparticle levels observed in the present study is associated with a lower increase in intracellular Ca^2+^ in platelets because microparticle release is a Ca^2+^-dependent process [6]. Polyphenols lead to inhibition of whole-blood aggregation suggesting a potential mechanism for the beneficial effects of polyphenols by the suppression of platelet-mediated thrombosis [42].

A significant correlation between endothelial microparticles and the loss of flow-mediated dilatation has been previously reported in obstructive sleep apnea [43] and in patients with end-stage renal failure [44] supporting the idea that these measurements are reliable indices of endothelial dysfunction. Thus, reduction of circulating microparticles may protect against endothelial dysfunction as evidenced by normalization of flow-mediated dilatation and intense vascular wall inflammation in our experimental model of hypertension. It is noteworthy that Provinols™ did not prevent the *in vitro* aldosterone-induced increase in microparticle production via shear stress. Although the in vitro conditions may not completely mimic the *in vivo* situation regarding extracellular Ca^2+^ and the level of shear stress, this result suggests that the *in vivo* effect of Provinols™ on microparticle levels was not dependent on shear stress.

The systolic hypertension observed in this model may be the result of the increase in stiffness of the vascular wall, as previously shown [2]. The significant increase in aortic dry weight and in cell proteins in aldosterone-salt rats reflects arterial wall hypertrophy, in accordance with previous studies [2], [45]. The finding of an increase in cell protein content but not of collagen is consistent with previous data showing an absence of collagen accumulation within the media in rats receiving aldosterone for 6 weeks [46]. The decreased elastin/collagen ratio and the increased cell protein content observed could partly explain the increased arterial stiffness, which is independent of any change in collagen.

In our study, in contrast to spironolactone, treatment with Provinols™ was not able to correct the hypertension. Previous studies have, however, reported antihypertensive effects of red wine polyphenol compounds, including Provinols™, in a rat DOCA-salt model of hypertension [47] and in other rat models; salt-sensitive, angiotensin II administration, NO-inhibition or the spontaneously hypertensive rat. However, in none of these models were the effects of Provinols™ compared to that of a mineralocorticoid receptor antagonist. In our model, systolic hypertension is dependent on the action of aldosterone on the mineralocorticoid receptor as demonstrated by the preventive effect of spironolactone. The hypotensive effect in control rats was also consistent with the permanent vascular tone driven by the microparticles. The absence of effect of Provinols™ on systolic hypertension could support the hypothesis that the effect in other models is related to interference with the renin angiotensin system, the NO pathway or the sympathetic nervous system [11], [12]. In agreement with this hypothesis, Provinols has no effect in control rats without changes in these pathways. Furthermore, spironolactone has been shown not to significantly lower systolic blood pressure in Ang II-infused rat [48]. This suggests that the effects of Provinols™ are not mediated via the mineralocorticoid receptor.

Aldosterone-salt-treated rats exhibited increased circulating and aortic NO levels. These effects were associated with an upregulation of iNOS in the vascular wall which was prevented by spironolactone and Provinols™. In line with this, we have reported in L-NAME hypertensive rats that the increase of iNOS expression in the aorta is reduced in rats treated with Provinols™ [12]. Moreover, we provide evidence that the effect of aldosterone is also associated with an increased oxidative stress, marked by increased aortic O_2_^−^ production, probably via activation of the NADPH oxidase pathway [49]. An increase in O_2_^−^ production leads to an enhancement of its interaction with NO and to the formation of the highly toxic ONOO^−^, as illustrated by the protein nitration in the aorta. Peroxynitrite is produced in large amounts here and has harmful effects in various tissues, especially in blood vessels. We demonstrate that spironolactone prevents the increase in O_2_^−^ production in the aorta as well as attenuating aldosterone-salt-induced ONOO^−^ production. Provinols™ prevented the increase in aortic O_2_^−^ production, probably due to their capacity to reduce aortic p22phox gene overexpression, observed in accordance with the work of Jimenez et al. [47] in DOCA-salt animals. This effect leads to reduced formation of ONOO^−^ and is also concomitant with a decrease in NFκB which is upstream to iNOS induction. Taken together, these data highlight both the anti-inflammatory effect and the decrease in oxidative stress induced by Provinols™ in the aorta, and therefore extend this beneficial action of polyphenols to aldosterone-salt-induced hypertension.

As for endothelial function, studies have shown that chronic treatment with aldosterone results in impaired acetylcholine-induced relaxation of the aorta in both normotensive and hypertensive rats via the production of cyclo-oxygenase 2 metabolites [5]. In small mesenteric arteries, we found that the response to acetylcholine was not altered whereas the vasodilator response to flow was reduced. This endothelial dysfunction in aldosterone-salt-treated rats was associated with a decrease in the NO component of shear stress. The mechanism by which aldosterone impairs flow-induced NO vasodilatation may result from the increase in oxidative stress that reduces endothelial NO bioavailability. This finding is in agreement with a previous report suggesting that increased NADPH-derived peroxide may be one possible mechanism for the reduced NO component of flow-mediated vasodilatation in SHR [50]. Provinols™ prevented endothelial dysfunction at the level of the microcirculation which is associated with enhanced endothelium-dependent relaxation resulting from augmented NO generation via increased eNOS activity and expression [9], [11], [12]. Also, we have reported that red wine polyphenols evoke endothelium-dependent relaxation associated with ERα-stimulation, c-Src/ERK1/2-mediated activation of eNOS, with consequent endothelial NO release [14]. Such a mechanism contributes probably to the beneficial effect of red wine polyphenols.

In conclusion, this study demonstrates that aldosterone-salt administration in rats is able to induce an increase in microparticles which is associated with endothelial dysfunction and vascular inflammation, and leads to the appearance of various oxidative stress markers. These arterial modifications could represent an early step in the development of hypertension. These changes were all reversed by spironolactone in all vascular territories, whereas the action of Provinols™, which is independent of mineralocorticoid receptor activity, preferentially affected the microcirculation and the NO pathway. The impact of red wine polyphenols on the production of microparticles and flow-induced dilatation provides new insights for future development of therapeutic strategies in aldosterone-mediated hypertension.

## Supporting Information

Figure S1 **Carotid artery incremental elastic modulus-wall stress (Einc- WS) curves.** **A.** Einc-WS curves from Control, Spironolactone (Spiro) and Provinols™ (Prov) rats (n = 5–9 for each group). **B.** Mean value of WS at 1500 kPa of Einc. Values are means ± SEM.
(TIF)

Figure S2 **NO production in aorta.** Quantification of the amplitude of NO-Fe(DETC)2 signal (amplitude per mg of dried sample Wds) in aorta from Control, Spironolactone (Spiro) and Provinols™ (Prov) rats (n = 6 for each group).
(TIF)

Table S1 **Effects of treatment by spironolactone (Spiro) or Provinols™ (Prov) on blood pressure, carotid diameter and organ weights in rats.**
(DOC)

## References

[1] Shibata S, Nagase M, Yoshida S, Kawachi H, Fujita T (2007). Podocyte as the target for aldosterone: roles of oxidative stress and Sgk1.. Hypertension.

[2] Lacolley P, Labat C, Pujol A, Delcayre C, Benetos A (2002). Increased carotid wall elastic modulus and fibronectin in aldosterone-salt-treated rats: effects of eplerenone.. Circulation.

[3] Brilla CG, Matsubara LS, Weber KT (1993). Anti-aldosterone treatment and the prevention of myocardial fibrosis in primary and secondary hyperaldosteronism.. J Mol Cell Cardiol.

[4] Savoia C, Touyz RM, Amiri F, Schiffrin EL (2008). Selective mineralocorticoid receptor blocker eplerenone reduces resistance artery stiffness in hypertensive patients.. Hypertension.

[5] Schiffrin EL (2006). Effects of aldosterone on the vasculature.. Hypertension.

[6] Martinez MC, Tual-Chalot S, Leonetti D, Andriantsitohaina R (2011). Microparticles: targets and tools in cardiovascular disease.. Trends Pharmacol Sci.

[7] Wang JM, Su C, Wang Y, Huang YJ, Yang Z (2009). Elevated circulating endothelial microparticles and brachial-ankle pulse wave velocity in well-controlled hypertensive patients.. J Hum Hypertens.

[8] Agouni A, Lagrue-Lak-Hal AH, Ducluzeau PH, Mostefai HA, Draunet-Busson C (2008). Endothelial dysfunction caused by circulating microparticles from patients with metabolic syndrome.. Am J Pathol.

[9] Diebolt M, Bucher B, Andriantsitohaina R (2001). Wine polyphenols decrease blood pressure, improve NO vasodilatation, and induce gene expression.. Hypertension.

[10] Andriambeloson E, Kleschyov AL, Muller B, Beretz A, Stoclet JC (1997). Nitric oxide production and endothelium-dependent vasorelaxation induced by wine polyphenols in rat aorta.. Br J Pharmacol.

[11] Bernatova I, Pechanova O, Babal P, Kysela S, Stvrtina S (2002). Wine polyphenols improve cardiovascular remodeling and vascular function in NO-deficient hypertension.. Am J Physiol Heart Circ Physiol.

[12] Pechanova O, Bernatova I, Babal P, Martinez MC, Kysela S (2004). Red wine polyphenols prevent cardiovascular alterations in L-NAME-induced hypertension.. J Hypertens.

[13] Lopez-Sepulveda R, Jimenez R, Romero M, Zarzuelo MJ, Sanchez M (2008). Wine polyphenols improve endothelial function in large vessels of female spontaneously hypertensive rats.. Hypertension.

[14] Chalopin M, Tesse A, Martinez MC, Rognan D, Arnal JF (2010). Estrogen receptor alpha as a key target of red wine polyphenols action on the endothelium.. PLoS One.

[15] Carlstrom M, Sallstrom J, Skott O, Larsson E, Persson AE (2007). Uninephrectomy in young age or chronic salt loading causes salt-sensitive hypertension in adult rats.. Hypertension.

[16] Park JB, Schiffrin EL (2001). ET(A) receptor antagonist prevents blood pressure elevation and vascular remodeling in aldosterone-infused rats.. Hypertension.

[17] Baron-Menguy C, Bocquet A, Guihot AL, Chappard D, Amiot MJ (2007). Effects of red wine polyphenols on postischemic neovascularization model in rats: low doses are proangiogenic, high doses anti-angiogenic.. Faseb J.

[18] Tual-Chalot S, Guibert C, Muller B, Savineau JP, Andriantsitohaina R (2010). Circulating microparticles from pulmonary hypertensive rats induce endothelial dysfunction.. Am J Respir Crit Care Med.

[19] Tardy Y, Meister J-J, Perret F, Brunner H, Ardity M (1991). Non-invasive estimate of the mechanical properties of peripheral arteries from ultrasonic and photoplethysmographic measurements.. Clin Phys Physiol Meas.

[20] Huang W, Alhenc Gelas F, Osborne-Pellegrin MJ (1998). Protection of the arterial internal elastic lamina by inhibition of the renin-angiotensin system in the rat.. Circ Res.

[21] Mulsch A, Mordvintcev P, Bassenge E, Jung F, Clement B (1995). In vivo spin trapping of glyceryl trinitrate-derived nitric oxide in rabbit blood vessels and organs.. Circulation.

[22] Kleschyov AL, Wenzel P, Munzel T (2007). Electron paramagnetic resonance (EPR) spin trapping of biological nitric oxide.. J Chromatogr B Analyt Technol Biomed Life Sci.

[23] Ohlmann P, Tesse A, Loichot C, Ralay Ranaivo H, Roul G (2005). Deletion of MLCK210 induces subtle changes in vascular reactivity but does not affect cardiac function.. Am J Physiol Heart Circ Physiol.

[24] Membre A, Wahl D, Latger-Cannard V, Max JP, Lacolley P (2008). The effect of platelet activation on the hypercoagulability induced by murine monoclonal antiphospholipid antibodies.. Haematologica.

[25] Lopez-Andres N, Fortuno MA, Diez J, Zannad F, Lacolley P (2010). Vascular effects of cardiotrophin-1: a role in hypertension?. J Hypertens.

[26] Di Zhang A, Nguyen Dinh Cat A, Soukaseum C, Escoubet B, Cherfa A (2008). Cross-talk between mineralocorticoid and angiotensin II signaling for cardiac remodeling.. Hypertension.

[27] Nakamura Y, Suzuki S, Suzuki T, Ono K, Miura I (2006). MDM2: a novel mineralocorticoid-responsive gene involved in aldosterone-induced human vascular structural remodeling.. Am J Pathol.

[28] Mercier N, Osborne-Pellegrin M, El Hadri K, Kakou A, Labat C (2006). Carotid arterial stiffness, elastic fibre network and vasoreactivity in semicarbazide-sensitive amine-oxidase null mouse.. Cardiovasc Res.

[29] Preston RA, Jy W, Jimenez JJ, Mauro LM, Horstman LL (2003). Effects of severe hypertension on endothelial and platelet microparticles.. Hypertension.

[30] Martinez MC, Tesse A, Zobairi F, Andriantsitohaina R (2005). Shed membrane microparticles from circulating and vascular cells in regulating vascular function.. Am J Physiol Heart Circ Physiol.

[31] Boulanger CM, Scoazec A, Ebrahimian T, Henry P, Mathieu E (2001). Circulating microparticles from patients with myocardial infarction cause endothelial dysfunction.. Circulation.

[32] Chahed S, Leroyer AS, Benzerroug M, Gaucher D, Georgescu A (2010). Increased vitreous shedding of microparticles in proliferative diabetic retinopathy stimulates endothelial proliferation.. Diabetes.

[33] Martin S, Tesse A, Hugel B, Martinez MC, Morel O (2004). Shed membrane particles from T lymphocytes impair endothelial function and regulate endothelial protein expression.. Circulation.

[34] Mesri M, Altieri DC (1998). Endothelial cell activation by leukocyte microparticles.. J Immunol.

[35] Priou P, Gagnadoux F, Tesse A, Mastronardi ML, Agouni A (2010). Endothelial dysfunction and circulating microparticles from patients with obstructive sleep apnea.. Am J Pathol.

[36] Meziani F, Tesse A, David E, Martinez MC, Wangesteen R (2006). Shed membrane particles from preeclamptic women generate vascular wall inflammation and blunt vascular contractility.. Am J Pathol.

[37] Williams TA, Verhovez A, Milan A, Veglio F, Mulatero P (2006). Protective effect of spironolactone on endothelial cell apoptosis.. Endocrinology.

[38] Martin S, Giannone G, Andriantsitohaina R, Martinez MC (2003). Delphinidin, an active compound of red wine, inhibits endothelial cell apoptosis via nitric oxide pathway and regulation of calcium homeostasis.. Br J Pharmacol.

[39] Jeong Y, Chaupin DF, Matsushita K, Yamakuchi M, Cameron SJ (2009). Aldosterone activates endothelial exocytosis.. Proc Natl Acad Sci U S A.

[40] Schafer C, Shahin V, Albermann L, Schillers H, Hug MJ H (2003). Intracellular calcium: a prerequisite for aldosterone action.. J Membr Biol.

[41] Lijnen P, Petrov V (1996). Cell membrane cation transport systems during aldorsterone antagonism.. J Cardiovasc Pharmacol.

[42] Freedman JE, Parker C, 3rd, Li L, Perlman JA, Frei B, et al (2001). Select flavonoids and whole juice from purple grapes inhibit platelet function and enhance nitric oxide release.. Circulation.

[43] Jelic S, Lederer DJ, Adams T, Padeletti M, Colombo PC (2009). Endothelial repair capacity and apoptosis are inversely related in obstructive sleep apnea.. Vasc Health Risk Manag.

[44] Boulanger CM, Amabile N, Guerin AP, Pannier B, Leroyer AS (2007). In vivo shear stress determines circulating levels of endothelial microparticles in end-stage renal disease.. Hypertension.

[45] Garwitz ET, Jones AW (1982). Aldosterone infusion into the rat and dose-dependent changes in blood pressure and arterial ionic transport.. Hypertension.

[46] Sun Y, Ramires FJ, Weber KT (1997). Fibrosis of atria and great vessels in response to angiotensin II or aldosterone infusion.. Cardiovasc Res.

[47] Jimenez R, Lopez-Sepulveda R, Kadmiri M, Romero M, Vera R (2007). Polyphenols restore endothelial function in DOCA-salt hypertension: role of endothelin-1 and NADPH oxidase.. Free Radic Biol Med.

[48] Zhao W, Ahokas RA, Weber KT, Sun Y (2006). Ang II-induced cardiac molecular and cellular events: role of aldosterone.. Am J Physiol Heart Circ Physiol.

[49] Callera GE, Touyz RM, Tostes RC, Yogi A, He Y (2005). Aldosterone activates vascular p38MAP kinase and NADPH oxidase via c-Src.. Hypertension.

[50] Zhou X, Bohlen HG, Miller SJ, Unthank JL (2008). NAD(P)H oxidase-derived peroxide mediates elevated basal and impaired flow-induced NO production in SHR mesenteric arteries in vivo.. Am J Physiol Heart Circ Physiol.

