# Supplementary figures and images for: Increased Microparticle Production and Impaired Microvascular Endothelial Function in Aldosterone-Salt-Treated Rats: Protective Effects of Polyphenols
Source: PLoS One. 2012 Jul 10;7(7):e39235. doi: 10.1371/journal.pone.0039235 (PMC3393732; doi:10.1371/journal.pone.0039235)

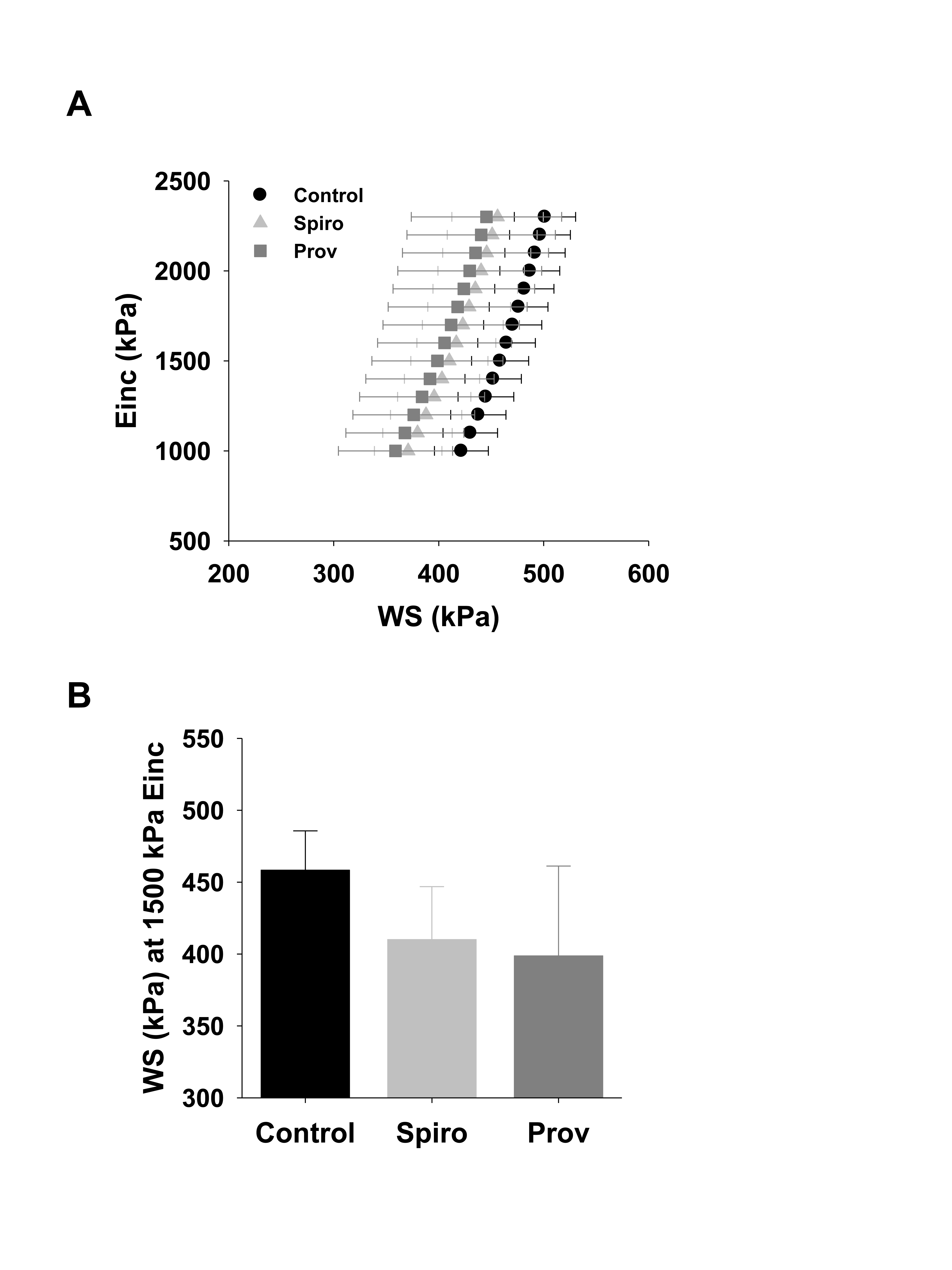

Supplement: Figure S1 — Carotid artery incremental elastic modulus-wall stress (Einc- WS) curves. A. Einc-WS curves from Control, Spironolactone (Spiro) and Provinols™ (Prov) rats (n = 5–9 for each group). B. Mean value of WS at 1500 kPa of Einc. Values are means ± SEM. (TIF) [file pone.0039235.s001.tif]

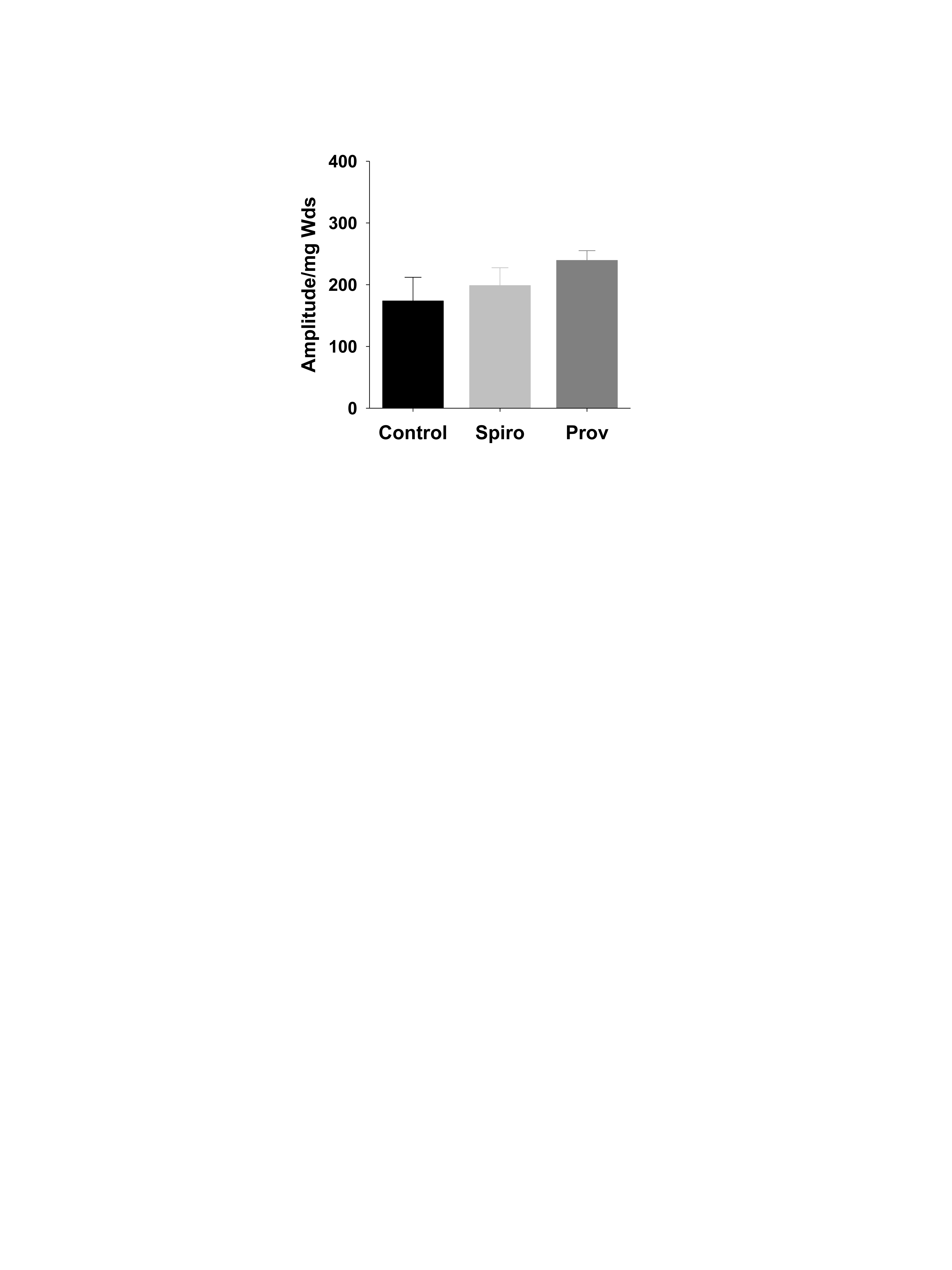

Supplement: Figure S2 — NO production in aorta. Quantification of the amplitude of NO-Fe(DETC)2 signal (amplitude per mg of dried sample Wds) in aorta from Control, Spironolactone (Spiro) and Provinols™ (Prov) rats (n = 6 for each group). (TIF) [file pone.0039235.s002.tif]
