# Supplementary material for: Increased Microparticle Production and Impaired Microvascular Endothelial Function in Aldosterone-Salt-Treated Rats: Protective Effects of Polyphenols
Source: PLoS One. 2012 Jul 10;7(7):e39235. doi: 10.1371/journal.pone.0039235 (PMC3393732; doi:10.1371/journal.pone.0039235)
Supplement: Table S1 — Effects of treatment by spironolactone (Spiro) or Provinols™ (Prov) on blood pressure, carotid diameter and organ weights in rats. (DOC) [file pone.0039235.s003.doc]

**Table S1**. **Effects of treatment by spironolactone (Spiro) or Provinols^TM^ (Prov) on blood pressure, carotid diameter and organ weights in rats.**

| **Group** | **Control** | **Spiro** | **Prov** |
| --- | --- | --- | --- |
| **N** | 27 | 5 | 11 |
| **Body weight (g)** | 420±6 | 374±7* | 403±3 |
| **DAP (mmHg)** | 117±4 | 95±7* | 110±4 |
| **SAP (mmHg)** | 161±4 | 136±7* | 154±6 |
| **MAP (mmHg)** | 132±4 | 109±7* | 124±3 |
| **PP (mmHg)** | 44±1 | 41±4 | 44±3 |
| **Diameter at MAP (mm)** | 1.30±0.03 | 1.40±0.03 | 1.22±0.05 |
| **Heart weight (g)** | 1.32±0.04 | 1.25±0.04 | 1.28±0.07 |
| **Kidney weight (g)** | 2.07±0.04 | 1.95±0.05 | 1.92±0.04* |

DAP: diastolic arterial pressure; SAP: systolic arterial pressure; MAP: mean arterial blood pressure; PP: pulse pressure. Values are means ± SEM * *P*<0.05 *vs* Control.
